# Physical exercise, mental health problems, and suicide attempts in university students

**DOI:** 10.1186/s12888-020-02583-3

**Published:** 2020-04-16

**Authors:** Michael Grasdalsmoen, Hege Randi Eriksen, Kari Jussie Lønning, Børge Sivertsen

**Affiliations:** 1 Department of Sport, Food and Natural Sciences, Western Norway University of Applied Sciences, Bergen, Norway; 2 The Norwegian Medical Association, Oslo, Norway; 3 The Student Welfare Association of Oslo and Akershus (SSiO), Oslo, Norway; 4 Department of Health Promotion, Norwegian Institute of Public Health, Bergen, Norway, Postboks 973 Sentrum, 5808 Bergen, Norway; 5 Department of Research & Innovation, Helse Fonna HF, Haugesund, Norway; 6 Department of Mental Health, Norwegian University of Science and Technology, Trondheim, Norway

**Keywords:** College students, Physical exercise, Mental health, Depression, Suicide attempt, Self-harm

## Abstract

**Background:**

Physical inactivity and mental health problems are both major public health concerns worldwide. Although several studies have demonstrated the health benefits of regular physical exercise, few epidemiological studies have investigated the nature of the association between different aspects of physical exercise and mental health, and little is known regarding the possible link to suicidality.

**Study aim:**

To examine the association between frequency, intensity, and duration of physical exercise and mental health problems, and to explore whether low levels of physical activity is related to self-harm and suicide attempts among college and university students.

**Methods:**

We employed data from the SHoT2018-study, a national health survey for higher education in Norway, in which 50,054 students aged 18–35 years participated. Physical exercise was assessed with three questions (frequency, intensity, and duration). Mental health problems were assessed with both a screening tool assessing psychological distress (Hopkins Symptom Checklist-25; HSCL-25) and self-reported depressive disorder (using a pre-defined list of conditions). Suicide attempts and self-harm were assessed with two items from the Adult Psychiatric Morbidity Survey.

**Results:**

Physical exercise was negatively associated with all measures of mental health problems and suicidality in a dose-response manner. The strongest effect-sizes were observed for frequency of physical exercise. Women with low levels of physical activity had a near three-fold increased odds of both scoring high on the HSCL-25, and self-reported depression, compared to women exercising almost every day. Even stronger effect-sizes were observed for men (ORs ranging from 3.5 to 4.8). Also, physical exercise duration and intensity were significantly associated with mental health problems, but with generally smaller ORs. Similarly, graded associations were also observed when examining the link to self-harm and suicide attempts (ORs ranging from 1.9 to 2.5).

**Conclusion:**

Given the demonstrated dose-response association between inactivity and both poor mental health, self-harm, and suicidal attempt, there is a need to facilitate college students to become more physically active. This is a shared responsibility that resides both on a political level and on the post-secondary institutions. The cross-sectional nature of the study means that one should be careful to draw firm conclusion about the direction of causality.

## Background

Despite overwhelming evidence showing extensive health benefits of physical exercise, a range of studies worldwide show that too few people engage in regular physical exercise. A 2018 Lancet paper, including 1.9 million participants from 168 countries, concluded that more than one in four adults worldwide fails to get the recommended levels of physical exercise (i.e., doing at least 150 min of moderate to vigorous physical activity [MVPA] per week) [1]. A recent national report from 2019, inviting all Norwegian university and college students, found even more disturbing results: less than one of four male, and less than one of five female students met the minimum recommended physical exercise criteria [2].

In addition to preventing a range of non-communicable diseases (NCDs), such as cardiovascular disease and type 2 diabetes [3], several studies have demonstrated the positive effects of physical exercise on mental health problems, especially depression [4, 5]. Global Burden of Disease studies have highlighted mental problems as one of the leading causes of disability globally, and depression is now considered the most disabling disorder worldwide measured in years lived with disability [6]. In addition to the immediate individual consequences of functional disability caused by depression (incl. Risk of poverty and social marginalization/isolation), the economic ramifications are devastating. According to recent estimates from the Norwegian authorities, the projected annual costs of depression in Norway are approximately 60–70 billion NOK (5–6 billion EUR). The economic costs of suicide, which most often occurs in the context of mental health problems, are also vast. For example, in Western Europe, each death by suicide costs at least 1.3 million EUR [7]. Due to these large negative consequences for both the individual and the society, national authorities have listed mental health problems as a top public health priority in the coming years [8].

There are surprisingly few epidemiological studies of mental health problems in college- and university students, despite being a crucial transitional period, with most mental disorders having an onset in late adolescence and early adulthood [9]. Some epidemiological studies have suggested that 12–50% of university- and college students meet the criteria for one or more common mental disorders [10–12], and the above-mentioned national survey in Norway recently reported an alarmingly high and increasing prevalence of both mental health problems [13] and suicidal ideation and non-suicidal self-harm (NSSH) [14] among college- and university students. Mental health problems are also directly associated with lower academic performance, which in turn, is associated with dropout in the short-term and loss of human capital for societies in the longer term [15]. Identifying risk and protective factors in this population may, therefore, have several benefits, given the substantial consequences that mental health problems have on subsequent educational, social, and economic outcomes.

Several studies have shown that physical exercise can generate several physiological changes and mechanisms in the body, which in turn may lower stress levels, or buffer the stress response which may protect against the negative health effects of stress [16–18], and improve mood and positive affect [19]. It has long been known that plasma levels of *endorphins* are elevated immediately following physical exercise, which in turn has been linked to feelings of euphoria among athletes [20]. Physical activity is also essential to maintain *mitochondrial biogenesis and function*, and prolonged inactivity has, therefore, been suggested to be central in developing pathophysiology of both somatic [21] and mental diseases [22]. Furthermore, physical exercise has been linked to the functioning of several *neurotransmitters* in the brain, including serotonin, dopamine, and noradrenaline, suggesting that physical exercise may act in the same way as the SSRI antidepressants [23]. Finally, physical exercise is important in maintaining proper functioning of the *hypothalamic-pituitary-adrenal (HPA) axis*, and several studies have demonstrated a dysfunction of the HPA axis in people suffering from mental disorders [24]. There are even studies suggesting that physical activity may have a comparable therapeutic effect on mental wellbeing, as to that of psychotherapy [4, 5, 25, 26].

In terms of potential mechanisms that might help explain the link between inactivity and poor mental health, other lifestyle factors and consequences of health behaviours (such as overweight/obesity [2, 27], alcohol use [28, 29], and sleep problems [30, 31]) have previously been linked to both physical exercise and mental wellbeing in young adulthood. These factors are important to account for when examining the association between physical exercise and mental health.

Despite evidence explaining the association between physical exercise and mental health, few large epidemiological studies are investigating the nature of the association between physical exercise and mental health. To our knowledge, no studies have explored the possible relation between physical exercise and suicidal ideation and NSSH. Based on these considerations, the aims of this large national study from 2018 was threefold: 1) to examine in detail the association between both the frequency, intensity and duration of physical exercise, and mental health problems among female and male university students, 2) to explore whether physical inactivity is related to NSSH and suicide attempt; and 3) to examine if any observed associations could partly be explained by sociodemographic or other lifestyle factors (such as BMI, alcohol use/problems and sleep duration).

## Methods

### Procedure

The SHoT study (Students’ Health and Wellbeing Study) is a large health survey comprising all fulltime college and university students aged 18 to 35 years in Norway. The main aim of the survey is to monitor the students’ mental and physical health, as well as psychosocial environment. The survey has been carried out three times (2010, 2014 and 2018), and the most recent wave (2018) was used in the current study. The survey data were collected electronically through an online platform. A total of 162,512 students fulfilled the inclusion criteria, of whom 50,054 (30.8%) students completed the online questionnaires [32].

### Independent variables

#### Physical exercise

The students were first presented with the following brief definition of physical exercise: “*With physical exercise, we mean that you, for example, go for a walk, go skiing, swim or take part in a sport*.” Physical exercise was then assessed using three sets of questions, assessing the average number of times exercising each week, and the average intensity and average hours each time [33]: 1*) “On an average week, how frequently do you physical exercise?”* (Never, Less than once a week, Once a week, 2–3 times per week, Almost every day); 2) *“If you do such physical exercise as frequently as once or more times a week: How hard do you push yourself?”* (I take it easy without breaking into a sweat or losing my breath, I push myself so hard that I lose my breath and break into a sweat, I push myself to near-exhaustion); and 3) *“How long does each session last?”* (Less than 15 min, 15–29 min, 30 min to 1 h, More than 1 h.”) This 3-item questionnaire has previously been used in the large population-based Nord-Trøndelag Health Study (the HUNT studies).

### Dependent variables

#### Mental health problems

Psychological distress was assessed using The Hopkins Symptoms Checklist (HSCL-25) [34], derived from the 90-itemSymptom Checklist (SCL-90), a screening tool designed to detect symptoms of anxiety and depression. It is composed of a 10-item subscale for anxiety, and a 15-item subscale for depression, with each item scored on a Likert scale from 1 (“*not at all*”) to 4 (“*extremely*”). The period of reference is the 2 weeks. Several factor structures and cut-offs for clinical levels have been proposed for the HSCL-25 [35, 36]. An investigation of the factor structure based on the SHoT2014 dataset showed that a uni-dimensional model had the best psychometric properties in the student population and not the original subscales of anxiety and depression [37]. We have chosen to follow this recommendation in the present study. Average scores on the HSCL-25 of ≥1.75 and < 2.00, and > 2.00, were used as cut-offs for identifying moderate and high levels, respectively, of psychological distress. Details on development of psychological distress in the SHoT waves were recently published by Knapstad et al. [13].

Self-reported depressive disorder was assessed by a pre-defined list adapted to fit this age-cohort. The list was based on a similar operationalization used in previous large population-based studies (the HUNT study [38]) and included several subcategories for most conditions/disorders (not listed here). For mental disorders, the list comprised the following specific disorders/group of disorders: ADHD, anxiety disorder, autism/Asperger, bipolar disorder, depression, PTSD (posttraumatic stress disorder), schizophrenia, personality disorder, eating disorder, Tourette’s syndrome, obsessive-compulsive disorder (OCD), and other. The list contained no definition of the included disorders/conditions. In the current study, only depressive disorder was included. The rationale for including both the HSCL-25 and the item of self-reported depression was to provide an assessment of overall symptom load (from the HSCL-25) and an indication of the presence of depression.

#### Self-harm and suicidal behavior

History of NSSH and suicide attempts were assessed with two items drawn from the Adult Psychiatric Morbidity Survey (APMS) [39]; *“Have you ever made an attempt to take your life, by taking an overdose of tablets or in some other way?”*, and *“Have you ever deliberately harmed yourself in any way but not with the intention of killing yourself? (i.e., self-harm)”* If respondents answered yes to any item, the timing of the most recent episode was assessed, using the following response options: “last week”, “past year”, “more than a year ago, but after I started studying at the university”, and “before I started studying at university”.

### Control variables

#### Sociodemographic information

All students indicated their age, sex, and relationship status. Economic activity was coded dichotomously by self-reported income per year (before deductions and tax, and not including scholarships/loans): “economically active” (income of > 10,000 NOK) versus “economically inactive” (< 10,000 NOK). Immigrant status was defined as either the student or any of his/her parents being born outside Norway.

#### Body mass index (BMI)

BMI was calculated as weight in kilos divided by squared height in metres, and then split into four categories: underweight (BMI < 18.5), normal weight (BMI 18.5–24.9), overweight (BMI 25.0–29.9) and obesity (BMI ≥ 30).

#### Sleep duration

Self-reported bedtime and rise time were indicated in hours and minutes (separately for weekdays and weekends), and the students’ time in bed was defined as the difference between these two time points. Sleep latency (defined as the amount of time it takes to go from being fully awake to sleeping) and nocturnal wake time after initial sleep onset, were also indicated. Sleep duration was thus operationalized as time in bed minus sleep latency and nocturnal wake time. Details of the sleep measures included in the SHoT2018 has been published elsewhere [40].

#### Alcohol-related problems

The Alcohol Use Disorders Identification Test (AUDIT) was used to measure alcohol-related problems. Developed by the World Health Organization for identifying risky or harmful alcohol use [41, 42], the AUDIT is a widely used instrument, and it has been validated in a wide range of ethnic groups. The AUDIT total score ranges from 0 to 40, a score of 8 or more is considered to indicate hazardous or harmful alcohol use (for a review, see [43]). More information about the AUDIT in the SHoT surveys has been published elsewhere [44].

### Statistical analyses

IBM SPSS Statistics 25 for Windows (SPSS Inc., Chicago, IL) was used for all statistical analyses. Pearson’s chi-squared tests were used to examine differences in the prevalence of psychological distress, depressive disorder, NSSH, and suicide attempt, by physical exercise stratified by gender. Logistic regression models were computed to obtain effect-size estimates, and results are presented as odds ratios (ORs) with 95% confidence intervals (95% CIs). Both crude and adjusted models were computed, the latter controlling for sociodemographics, body-mass index, alcohol use, and problems and sleep duration (entered in one block). Estimated marginal means (EMM) was also computed to examine physical exercise frequency against the HSCL-25 average score, adjusting for the above confounders. There was generally little missing data (*n* < 250 of 50,054 on the three physical exercise items), and the missing values were handled using listwise deletion.

### Ethics

The study was approved by the Regional Committee for Medical and Health Research Ethics in Western Norway (no. 2017/1176 [SHOT2018]). All participants provided electronic informed consent after receiving a detailed introduction to the study.

## Results

### Psychological distress

**Fig. 1:**
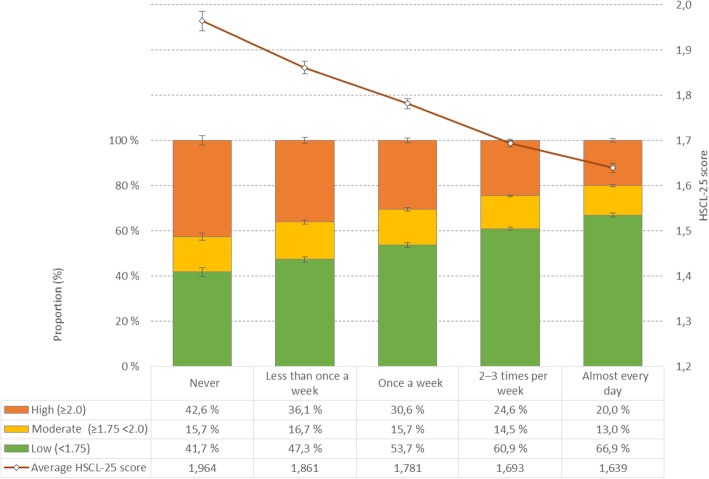
Psychological distress (HSCL-25) presented in categories (bars) and average score (red line) by physical exercise frequency. Average scores represent estimated marginal means (EMM), adjusted for socio-demographics, body-mass index, alcohol use and problems and sleep duration. Error bars represent 95% confidence intervals

The students’ level of psychological distress was negatively associated with physical exercise in a dose-response manner. As detailed in Tables 1, 67% of women who reported that they, on average, never physically exercised during the week scored above the cut-off of 1.75 on the HSCL-25, indicating a moderate level of psychological distress, compared to 40% of women exercising almost every day (OR = 2.95, 95% CI: 2.61–3.35). A similar trend was observed in men, where 46% of men who never physical exercised scored above the same cut-off, compared to 19% in men exercising almost every day (OR = 3.53, 95% CI: 3.03–4.11). Similar patterns and associations to psychological distress were observed for both the *intensity* and *duration* of the physical exercise (see Table 1 for details). The ORs were generally larger for men than women, with significant gender interactions for the *frequency* and *duration* variables (*p* < .001), but not *intensity* (*p* = .35). Adjusting for sociodemographics, BMI, alcohol use/problems, and sleep duration only marginally attenuated the ORs. As displayed in Fig. 1, students who physical exercised less frequently had a significantly higher average HSCL score, compared to students who physical exercised more often.

**Table 1:** Association between physical exercise and psychological distress (HSCL-25) in male and female university and college students in the SHoT study, Norway, 2018

	Psychological distress (HSCL average score > 1.75)							
			Unadjusted model			Adjusted model^a^		
	n	%	OR	95%CI-	95%CI+	OR	95%CI-	95%CI+
**Women**								
**Physical exercise frequency**								
*Never*	864	66.6%	2.96	2.61	3.35	2.63	2.29	3.03
*Less than once a week*	2438	60.1%	2.24	2.07	2.42	2.04	1.88	2.23
*Once a week*	3012	53.0%	1.67	1.56	1.79	1.64	1.52	1.77
*2–3 times per week*	7022	44.9%	1.21	1.14	1.28	1.18	1.11	1.26
*Almost every day*	3077	40.3%	1.00	–	–	1.00	–	–
**Physical exercise intensity**								
*I take it easy without breaking into a sweat or losing my breath*	3539	56.8%	1.46	1.33	1.60	1.57	1.42	1.73
*I push myself so hard that I lose my breath and break into a sweat*	10,608	44.5%	0.89	0.82	0.96	0.93	0.85	1.01
*I push myself to near-exhaustion*	1332	47.4%	1.00	–	–	1.00	–	–
**Physical exercise duration**								
*Less than 15 min*	324	61.7%	1.96	1.64	2.35	2.07	1.69	2.53
*15–29 min*	1875	56.8%	1.60	1.48	1.73	1.71	1.56	1.86
*30 min to 1 h*	8634	46.0%	1.04	0.99	1.09	1.10	1.04	1.16
*More than 1 h*	4657	45.1%	1.00	–	–	1.00	–	–
**Men**								
**Physical exercise frequency**								
*Never*	404	45.8%	3.53	3.03	4.11	3.12	2.64	3.69
*Less than once a week*	811	38.5%	2.62	2.33	2.95	2.45	2.15	2.78
*Once a week*	615	28.4%	1.66	1.47	1.87	1.64	1.44	1.87
*2–3 times per week*	1444	23.9%	1.31	1.19	1.45	1.30	1.17	1.44
*Almost every day*	784	19.3%	1.00	–	–	1.00	–	–
**Physical exercise intensity**								
*I take it easy without breaking into a sweat or losing my breath*	730	35.0%	1.63	1.43	1.85	1.70	1.48	1.96
*I push myself so hard that I lose my breath and break into a sweat*	2309	23.5%	0.93	0.84	1.03	0.99	0.89	1.11
*I push myself to near-exhaustion*	603	24.8%	1.00	–	–	1.00	–	–
**Physical exercise duration**								
*Less than 15 min*	118	41.4%	2.53	1.98	3.22	2.46	1.88	3.23
*15–29 min*	406	32.3%	1.70	1.49	1.94	1.85	1.60	2.13
*30 min to 1 h*	1599	27.2%	1.34	1.23	1.45	1.35	1.23	1.47
*More than 1 h*	1513	21.9%	1.00	–	–	1.00	–	–

^a^ Adjusted for sociodemographics, body-mass index, alcohol use and problems and sleep duration

### Depressive disorder

Self-reported depression was also negatively associated with physical exercise frequency in a dose-response manner; the more infrequent the students physical exercised, the higher the prevalence of depression. As detailed in Table 2, one in four female students who never reported exercising had depression, compared to one in ten female students exercising almost every day (OR = 2.99, 95% CI: 2.58–3.46). An even stronger association was observed in men, with a corresponding OR of 4.86 (95% CI: 3.88–6.09). As detailed in Table 2, similar graded patterns were observed for both the *intensity* and *duration* of the physical exercise, but there were no significant gender interactions for these two variables. Adjusting for sociodemographics, BMI, alcohol use/problems and sleep duration did not, or only slightly, attenuate the ORs.

**Table 2:** Association between physical exercise and self-reported depression in male and female university and college students in the SHoT study, Norway, 2018

	Self-reported depression							
			Unadjusted model			Adjusted model^a^		
	n	%	OR	95%CI-	95%CI+	OR	95%CI-	95%CI+
**Women**								
**Physical exercise frequency**								
*Never*	320	24.7%	2.99	2.58	3.46	2.53	2.15	2.98
*Less than once a week*	720	17.7%	1.97	1.77	2.20	1.70	1.50	1.91
*Once a week*	802	14.1%	1.50	1.35	1.67	1.42	1.27	1.59
*2–3 times per week*	1702	10.9%	1.12	1.02	1.22	1.09	0.99	1.20
*Almost every day*	754	9.9%	1.00	–	–	1.00	–	–
**Physical exercise intensity**	320							
*I take it easy without breaking into a sweat or losing my breath*	1060	17.0%	1.57	1.37	1.79	1.47	1.27	1.69
*I push myself so hard that I lose my breath and break into a sweat*	2585	10.8%	0.93	0.83	1.06	0.92	0.81	1.05
*I push myself to near-exhaustion*	324	11.5%	1.00	–	–	1.00	–	–
**Physical exercise duration**								
*Less than 15 min*	113	21.5%	2.29	1.84	2.84	2.14	1.68	2.73
*15–29 min*	546	16.5%	1.65	1.48	1.84	1.56	1.39	1.77
*30 min to 1 h*	2201	11.7%	1.11	1.03	1.20	1.10	1.01	1.20
*More than 1 h*	1106	10.7%	1.00	–	–	1.00	–	–
**Men**								
**Physical exercise frequency**								
*Never*	164	18.5%	4.86	3.88	6.09	4.08	3.19	5.20
*Less than once a week*	254	12.0%	2.92	2.40	3.56	2.58	2.09	3.18
*Once a week*	180	8.3%	1.93	1.56	2.39	1.87	1.49	2.34
*2–3 times per week*	401	6.6%	1.51	1.26	1.81	1.45	1.20	1.76
*Almost every day*	182	4.5%	1.00	–	–	1.00	–	–
**Physical exercise intensity**								
*I take it easy without breaking into a sweat or losing my breath*	240	11.5%	1.95	1.58	2.41	1.85	1.47	2.33
*I push myself so hard that I lose my breath and break into a sweat*	620	6.3%	1.01	0.84	1.21	1.05	0.86	1.28
*I push myself to near-exhaustion*	152	6.2%	1.00	–	–	1.00	–	–
**Physical exercise duration**								
*Less than 15 min*	40	13.9%	2.67	1.88	3.79	2.35	1.61	3.45
*15–29 min*	149	11.8%	2.21	1.81	2.70	2.25	1.82	2.78
*30 min to 1 h*	426	7.2%	1.29	1.12	1.48	1.28	1.11	1.49
*More than 1 h*	397	5.7%	1.00	–	–	1.00	–	–

^a^ Adjusted for sociodemographics, body-mass index, alcohol use and problems and sleep duration

### Self-harm and suicidal behavior

Having a history of self-harm and suicidal behavior was significantly more common among students who physical exercised less. Similar to the findings for mental health problems and depression, the strongest associations among women were observed for physical exercise frequency, with students never exercising having an approximately 2 to 2.5-fold increased odds of having a history of self-harm and suicidal behavior, compared to students exercising almost every day. For men, the strongest association was observed for the *duration* of the physical exercise. However, in contrast to the findings for mental health problems and depression, there was a U-shaped association between physical exercise intensity and women’s NSSH and suicide attempt, as well as the association between physical exercise duration and suicide attempt in women (see Table 3 for details). The group with moderate physical exercise intensity had lower odds of NSSH and suicide attempt compared to the groups with the highest *and* lowest intensity. Similarly, female students exercising 30 min to one hour had *lower* odds of suicide attempt, compared to those with more than one hour physical exercise. Also here, adjusting for potential confounders had little, or no, impact on the magnitude of the ORs.

**Table 3:** Association between physical exercise and non-suicidal self-harm (NSSH) and suicide attempt in male and female university and college students in the SHoT study, Norway, 2018

	Non-suicidal self-harm (NSSH)								Suicide attempt							
			Unadjusted model			Adjusted model^a^					Unadjusted model			Adjusted model^a^		
	n	%	OR	95% CI-	95%CI+	OR	95%CI-	95%CI+	n	%	OR	95%CI-	95%CI+	OR	95%CI-	95%CI+
**Women**																
**Physical exercise frequency**																
*Never*	447	34.5%	1.91	1.68	2.17	1.78	1.54	2.05	30	2.3%	2.53	2.05	3.13	1.99	1.57	2.51
*Less than once a week*	1200	29.6%	1.53	1.40	1.66	1.39	1.27	1.53	65	1.6%	1.58	1.33	1.86	1.24	1.03	1.49
*Once a week*	1417	25.0%	1.21	1.11	1.31	1.17	1.07	1.27	51	0.9%	1.08	0.91	1.27	0.96	0.81	1.15
*2–3 times per week*	3396	21.7%	1.01	0.94	1.08	0.95	0.89	1.02	142	0.9%	0.97	0.85	1.11	0.89	0.77	1.03
*Almost every day*	1651	21.6%	1.00	–	–	1.00	–	–	67	0.9%	1.00	–	–	1.00	–	–
**Physical exercise intensity**																
*I take it easy without breaking into a sweat or losing my breath*	1757	28.2%	1.21	1.09	1.34	1.27	1.13	1.41	83	1.3%	1.13	0.94	1.37	1.06	0.86	1.30
*I push myself so hard that I lose my breath and break into a sweat*	5193	21.8%	0.86	0.78	0.94	0.87	0.79	0.96	201	0.8%	0.67	0.57	0.80	0.69	0.58	0.83
*I push myself to near-exhaustion*	688	24.5%	1.00	–	–	1.00	–	–	41	1.5%	1.00	–	–	1.00	–	–
**Physical exercise duration**																
*Less than 15 min*	150	28.8%	1.38	1.13	1.68	1.35	1.08	1.68	10	1.9%	1.49	1.05	2.11	1.20	0.80	1.81
*15–29 min*	972	29.5%	1.43	1.31	1.56	1.48	1.34	1.63	41	1.2%	1.34	1.13	1.58	1.28	1.07	1.54
*30 min to 1 h*	4181	22.3%	0.98	0.92	1.04	1.00	0.94	1.06	167	0.9%	0.85	0.75	0.95	0.85	0.75	0.97
*More than 1 h*	2338	22.7%	1.00	–	–	1.00	–	–	107	1.0%	1.00	–	–	1.00	–	–
**Men**																
**Physical exercise frequency**																
*Never*	134	15.2%	1.92	1.55	2.38	1.66	1.31	2.09	18	2.0%	2.01	1.36	2.95	1.85	1.23	2.80
*Less than once a week*	306	14.5%	1.82	1.54	2.14	1.66	1.39	1.97	30	1.4%	2.13	1.59	2.86	2.05	1.50	2.81
*Once a week*	234	10.8%	1.29	1.09	1.54	1.22	1.01	1.47	21	1.0%	1.49	1.09	2.05	1.36	0.96	1.92
*2–3 times per week*	584	9.6%	1.14	0.99	1.31	1.10	0.95	1.28	56	0.9%	1.24	0.96	1.61	1.20	0.90	1.59
*Almost every day*	347	8.5%	1.00	–	–	1.00	–	–	20	0.5%	1.00	–	–	1.00	–	–
**Physical exercise intensity**																
*I take it easy without breaking into a sweat or losing my breath*	313	15.0%	1.67	1.40	2.01	1.62	1.33	1.96	30	1.4%	1.66	1.23	2.25	1.63	1.17	2.26
*I push myself so hard that I lose my breath and break into a sweat*	920	9.3%	0.98	0.84	1.14	1.00	0.85	1.17	72	0.7%	0.78	0.60	1.01	0.80	0.60	1.07
*I push myself to near-exhaustion*	232	9.6%	1.00	–	–	1.00	–	–	25	1.0%	1.00	–	–	1.00	–	–
**Physical exercise duration**																
*Less than 15 min*	52	18.2%	2.28	1.67	3.12	2.11	1.50	2.97	9	3.1%	2.33	1.37	3.94	1.61	0.86	3.03
*15–29 min*	177	14.1%	1.68	1.41	2.01	1.80	1.49	2.18	11	0.9%	1.72	1.26	2.36	2.03	1.46	2.83
*30 min to 1 h*	619	10.5%	1.21	1.07	1.36	1.19	1.05	1.35	54	0.9%	1.23	0.99	1.52	1.32	1.05	1.66
*More than 1 h*	615	8.9%	1.00	–	–	1.00	–	–	53	0.8%	1.00	–	–	1.00	–	–

^a^ Adjusted for sociodemographics, body-mass index, alcohol use and problems and sleep duration

### Discussion

This large national health survey from 2018, inviting all Norwegian full-time university- and college students in the age range 18–35, has several interesting discoveries. Physical exercise was negatively associated with all measures of mental health problems and suicidality in a dose-response manner. The strongest ORs were observed for the *frequency* of physical exercise, with women never exercising having a near three-fold increased odds of both scoring above the clinical cut-off on the HSCL-25, and of self-reported depression, compared to women exercising almost every day. Even stronger associations were observed for men. Also, physical exercise *duration* and *intensity were* significantly associated with mental health problems. Similarly, graded associations were also observed when examining the association to self-harm and suicide attempts. However, care should be taken when interpreting the direction of associations from this cross-sectional survey.

The current study extends on the findings from the recent and very large US study of 1.2 million individuals, who also found a strong link between physical exercise and mental health [45]. However, in contrast to the study by Chekroud et al., who failed to find a dose-response relationship between the two variables [45], the current study *did* find that the more physical exercise, the better, both in terms of physical exercise frequency and duration. While we cannot explain with certainty why these findings differ, there are some important methodological differences between the two studies that should be noted. First, whereas the study Chekroud et al. used a single item assessing “mental health burden” as the sole outcome variable, the current study used several well-validated questionnaires, including symptoms of anxiety and depression, self-reported depression, and non-suicidal self-harm and suicide attempt. The dose-response pattern was observed for all these measures. Also, the physical exercise measures differed somewhat between these two studies, with the Chekroud study focusing more on *type* of physical exercise, while the current study included three items previously validated against VO_2_max [33, 46], and with the International Physical Activity Questionnaire [47]. More research is needed to establish if, and under what circumstances, there is a linear or U-shaped association between physical exercise and mental health. The assertion made by Chekroud and colleagues that those with the highest levels of physical exercise also reported poorer mental health, have sparked some discussion, with researchers claiming that this interpretation is both unwarranted and unfortunate [48]. Of note, it should be mentioned that also the current study, when examining NSSH and suicide attempts among women (but not men), found a U-shaped association between physical exercise intensity and these outcomes. It is difficult to speculate why these findings differ from the overall pattern of linear associations.

Whereas the current epidemiological study was not designed to examine the pathways or mechanisms involved in the physical exercise-mood link, the inclusion of several potential confounders might shed some light on this topic. However, adjusting for both sociodemographics, health behaviors and sleep duration did not or only slightly attenuate the observed associations. In terms of biological mechanisms involved in the physical exercise-mood association, a recent meta-analysis [49] suggested that the anti-depressant effect of physical exercise is not merely caused by a short-term boost in endorphin levels [20], but may also help stimulate brain function on a broader level. Physical exercise seems to be important in the maintenance of mitochondrial functioning, and prolonged inactivity is likely to be central in developing pathophysiology of ill health [21, 22]. Still, our knowledge about why physical exercise seems to have such a strong anti-depressant effect remains limited, and there is a clear need for well-designed epidemiological and physiological research that may further disentangle the involved mechanisms. One approach may be to examine the social aspects of physical exercise, given the well-known link between loneliness and poor mental health [50]. Similarly, the close link between physical activity and both sleep quality and quantity [51], may warrant more focus also in relation to their effect on mood. A recent paper of adolescents suggested that sleep may indeed mediate the association between physical activity and mental health problems [52].

The findings have some important public health implications, as they call attention to both the high prevalence of mental health problems among students taking higher education, as well as the disturbingly high proportion of young adults failing to meet the recommended levels of weekly physical exercise. In terms of potential measures to tackle the rising mental health problems currently observed in college- and university students, the low levels of physical activity in this population may be one of the more modifiable factors that may be fruitful to target. Regarding both prevention and treatment of mental health problems, physical exercise and in particular outdoor activities have been suggested as particularly beneficial, with a recent systematic review showing that outdoor, nature-based exposure has a positive effect on several emotional parameters, related to stress relief [53].

Despite conflicting findings regarding the existence of a dose-response relationship between physical exercise and mood, most will agree that even a small increase in physical exercise from inactivity is beneficial. Although both educational institutions and student welfare organizations try hard to encourage and facilitate their students to take part in a wide range of sports, physical exercise and outdoor activities, the current results suggest that increase efforts are warranted. One new approach may be to adopt strategies from e.g. high schools, where physical exercise is an integrated element in the typical school day. Furthermore, in terms of planning new campuses, prioritizing good and safe cycle paths and walkways may be a good strategy [54]. Although there is limited evidence of the effectiveness of broad awareness campaigns to increase the public’s physical activity, there are studies suggesting that various forms of personalized media messages can be used to raise awareness, increase knowledge, and motivate a population to be more physically active [55].

An important limitation of the study is the cross-sectional design, which makes it difficult to evaluate the directionality between mental health and physical exercise. Although there is substantial evidence showing that regular physical exercise has a positive effect on mood [49], there are also studies showing that the association is likely to be bidirectional. Longitudinal studies have found depressive symptomatology to predict subsequent lower activity levels [56], and there are also plausible mechanisms which may explain how symptoms of depression may lead to inactivity. These include e.g. low energy levels or apathy [57], psychomotor retardation and anhedonia [58], and social isolation which in turn may reduce the motivation to be active [59]. However, there are far fewer prospective studies examining this directionality, and future studies should seek to include analysis exploring further the bidirectionality between physical exercise and mental health. Another limitation is the response rate of 31%, and we also had limited information regarding non-responders. It is possible the web-based data collection contributed to the modest response rate, as electronic platforms has been found yield lower participation rates compared to traditional approaches, and especially mixed mode designs [60, 61]. Another limitation is that while sleep duration was included as a confounding variable in this study, we did not control for subjective sleep quality, which is generally closely associated with mental health problems [62]. A final limitation regarding the physical exercise measure is that it is more accurate to say that we measured the students’ *perceived* intensity, as less fit students may feel more exhausted by an intensity level that a fit person will feel comfortable with. Furthermore, the physical exercise instrument only assesses the average exercise level, and students with varying exercise regimes may find it difficult to answer accurately. However, previous validation studies [33, 46] of the physical exercise items have demonstrated strong correlations between the questionnaire responses and direct measurement of VO_2_max during maximal work on a treadmill, with ActiReg [63], an instrument that measures PA and energy expenditure (EE), and with the International Physical Activity Questionnaire [47, 64].

The strengths of the study include the large and heterogeneous sample, as most previous studies in this field have examined white, young and female undergraduates [65]. Other strengths include well-validated instruments of both physical exercise, mental health problems, NSSH and suicide attempts, in addition to the extensive list of potential confounders.

Given the demonstrated dose-response link between inactivity and both poor mental health, self-harm, and suicidal attempt, there is a strong need to facilitate our college- and university students to become more physically active, a shared responsibility that resides both on a political level, welfare organizations, and on the education institutions.

## Data Availability

The SHoT dataset is administrated by the NIPH. Approval from a Norwegian regional committee for medical and health research ethics [https://helseforskning.etikkom.no] is a pre-requirement. Guidelines for access to SHoT data are found at [https://www.fhi.no/en/more/access-to-data].

## Abbreviations

PA: Physical activity
MVPA: Moderate to vigorous physical activity
NSSH: Non-suicidal self-harm
SHOT: Students’ health and wellbeing study
NCDs: Noncommunicable diseases
HPA: Hypothalamic pituitary-adrenal
BMI: Body-mass index
HSCL-25: The Hopkins symptoms checklist-25
EMM: Estimated marginal means
WHO: World health organization
